# Towards miniaturized electrochemical sensors for monitoring of polychlorinated biphenyls

**DOI:** 10.12688/openresafrica.13983.1

**Published:** 2023-04-14

**Authors:** Elizabeth Nthambi Ndunda, Moses Mutiso Mwanza

**Affiliations:** 1 Department of Physical Sciences, School of Pure and Applied Sciences, Machakos University, Machakos, Machakos County, Kenya

**Keywords:** Electrochemical sensors, Biomimetic sensors, PCBs, MIPs, environmental pollution, screen-printed electrodes

## Abstract

Pollution of our environment as a result of industrialization and other human activities is a growing concern due to the harmful effects of most chemicals that are released into the environment. Of particular interest are the persistent organic pollutants (POPs) that are reported to be toxic and build up in the environment due to their persistence. Among the POPs are polychlorinated biphenyls (PCBs), which were widely used in the past in various applications ranging from additives in pesticides to dielectric fluids in electrical equipment. As a way of protecting the one health trilogy (environment, human and animal health), their determination in the environment is a paramount call that has seen researchers continue to provide advanced technologies towards achieving this goal. These technologies involve the conventional gold standard gas chromatography systems coupled to sensitive detectors that can detect trace level concentrations. They have come in handy in monitoring of PCBs but their application for routing monitoring may not be sustainable because of the cost of operation associated with them and the need for experts to run the equipment. As a result, there is need for affordable systems that are still able to achieve the required sensitivity for routine monitoring and real-time data acquisition. Sensor systems fit very well in this category since they can be miniaturized for affordability and portray many other desirable features. PCBs as environmentally relevant environmental pollutants have received minimal attention with regards to sensor development and this review highlights the efforts that have been made so far. It provides in-depth discussions on electrochemical sensors and the various modifications that have been employed to date to achieve detection of PCBs at low concentrations as well as the future prospects in remote and routine monitoring.

## Introduction

Polychlorinated biphenyls (PCBs) are manmade industrial chemicals that are very stable, portraying resistance to extreme temperature and pressure ^1^. These properties rendered them useful in a wide range of applications mostly in electrical equipment and other uses in lubricants, hydraulic fluids and plasticizers in plastics and paints ^2^. They were produced majorly in the period between 1930s and 1970s being sold as mixtures under different trade names, with the common one being Aroclor ^1, 3^. As a result of their widespread usage, PCBs found their way into the environment through spills and leakage from electrical and other equipment during their production and transport as well as improper disposal and storage ^4^. Furthermore, there are reports that these chemicals continue to be released through improper dumping of PCBs containing materials as well as open burning and incineration of both municipal and industrial waste ^5^. It is estimated that out of the 1.3 million tons of PCBs that were produced globally, more than half have been released into the environment ^6^.

Once in the environment, PCBs are transported over long distances owing to their long range transport property where they bind strongly to soil and sediment that act as secondary sources of PCBs through volatilizations ^7^. Polychlorinated biphenyls have been reported in various environmental media ranging from indoor air ^8, 9^, water and sediment ^10, 11^ and soil ^12^. Of great concern is that these chemicals enter into the food chain where human beings are exposed mainly through the diet and breathing contaminated air, having been detected in human tissue including blood ^13^, adipose tissue ^14^, liver and kidney ^15^. Reported health effects include potential to cause cancer ^16^, disruption of the endocrine system, reduced intellectual quotient (IQ) and diabetes ^17^ among others. PCBs have the potential to bioaccumulate in fatty tissues of organisms ^4^ where they may reach toxic levels; meaning continued exposure even at low concentration in the environment may result into long term health effects.

Measures taken to reduce PCBs in the environment and body burden include adoption of the Stockholm convention on POPs in 2004 that placed restrictions on production and use of PCBs and calls for continued surveillance inform of environmental monitoring. Routine reporting on contamination of the environment with PCBs serves as a way of tracking pollution sources with a view to eliminate them ^18^. Their separation and detection in the environmental samples has been achieved by use of chromatographic techniques with electron capture detector (GC-ECD) or mass spectrometer as the detection systems ^10, 19, 20^ and recently the sensitive and selective high and low resolution mass-spectrometry (HR/LS-MS) ^21, 22^. These techniques are highly sensitive and have been able to provide the required information albeit with high cost implications that do not favor routine monitoring. As a way of addressing these challenges and achieve routine as well as remote monitoring of PCBs, other analytical formats have been introduced. Bioassays that utilize living organisms to detect or determine the toxicity of contaminants are some of the formats that have been reported ^23^. Chemical-activated luciferase gene expression (CALUX) bioassay and enzyme-linked immunosorbent assays (ELISA) have been reported as providing results comparable to the conventional methods ^24, 25^. The technicalities of working with biologically generated elements in bioassays has seen the introduction of robust chemical sensors that aim to achieve the required merits of sensitivity, selectivity, affordability and fast response time ^23^. This review therefore focuses on versatile electrochemical sensors, which are the ones that have been majorly applied in environmental monitoring of many chemical contaminants including PCBs owing to their sensitivity, selectivity, ease of integrating with nanomaterials, miniaturization potential and cost-effectiveness.

## Electrochemical sensors

In search of the best alternatives to the expensive conventional equipment used in detection of PCBs, sensors have been perceived as better placed. Their attractive properties that include ease of fabrication, low cost and the possibility of providing real-time information and remote sensing ^23^ make them better options to pursue. Chemical sensors transform chemical information into an analytically useful signal that can be measured. This is achieved by having a sensing layer that interacts with the analyte to produce a chemical change that is eventually transformed into a readable electronic signal by a transducer. Based on the transduction, there are six techniques: electrochemical, optical, mechanical, magnetic, thermometric and microgravimetric, with electrochemical being among the most frequently used techniques as it displays high sensitivity in fabrication of biosensors and chemosensors ^26^. Electrochemical sensors consist of a recognition element or chemically selective layer coupled an electrochemical transducer to provide real time information through the interaction with a solution that contains a target analyte. There are four main electrochemical methods depending on the electrochemical signal transduction. They include: potentiometric methods that relate the change in potential to the concentration of the analyte; amperometric that determine the change in current due to oxidation or reduction of electroactive species at a constant potential or varied potential in the case of voltammetry and electrochemical impedance spectroscopy (EIS) that measures the change in current or the impedance upon an applied sinusoidal voltage in a given frequency range ^26, 27^.

Electrochemical techniques rely on solid electrodes made of gold, carbon, and platinum as inert materials. These electrodes act as support surfaces for the immobilization of the recognition element as well as transducing device. In most cases, the electrodes are modified to increase the sensitivity and selectivity to a particular analyte through immobilization of a selective layer on the transducer surface via ex situ or in situ methods such as spin coating and electropolymerization, respectively ^28^. The latter offers the opportunity for film thickness control, which is an attractive aspect for generation of reproducible results, and the films produced do not require further processing ^28^. The target analytes in these electrochemical sensors are expected to be electroactive undergoing redox reaction upon an applied potential ^28, 29^ though, indirect approaches have been applied for species that are not electroactive by utilizing an electroactive probe to obtain the electrochemical response ^30, 31^. PCBs are not electroactive and therefore redox probes such as ferricyanide/ferrocyanide [Fe(CN)6] ^4−/3−^ ^32^ and ferrocene ^33^ have been utilized. In such case, the current response of the redox probe varies inversely with the concentration of the analyte since the diffusion of the redox probe to the surface of the electrode is inhibited by the high concentration of the analyte resulting into a decreased signal ^34^.

So far the reported electrochemical sensors for detection of PCBs are based on modifications of the sensing layer using pyrenecyclodextrin as a composite with carbon nanotubes, reduced graphene oxide or gold nanoparticles. Cyclodextrin form a host-guest complex with PCBs thus increasing the selectivity of the sensors, while graphene, carbon nanotubes and nanoparticles increase the electrode conductivity enhancing the sensor signal and the sensitivity of the electrodes ^33, 35, 36.^ Detection of PCBs in these electrochemical sensors has been based on differential pulse voltammetry (DPV), square wave voltammetry (SWV) and electrochemical impedance spectroscopy (EIS) techniques that are sensitive ^37^. An electrochemical sensor based on single-walled carbon nanotube/pyrenecyclodextrin (SWCNT/ PyCD) hybrid was reported to detect PCB-77 at nM range ^35^. Another sensor whose recognition element was a composite of reduced graphene oxide and β-cyclodextrin polymer (rGO/β-CDP) was reported to detect a mixture of Aroclors. Its application in determination of PCBs in sediment core provided a better detection limit of 0.5 pM compared to 9.2 pM for the GC-ECD, thus providing a tool that can possibly be designed for determination of PCBs at trace levels ^33^. A similar sensor that incorporated gold nanoparticles achieved a detection limit of 0.028 µΜ for PCB-77 and recoveries of 94–106% for environmental water samples. The sensor was also reported to be responsive to PCB 126 and 169, which are coplanar PCBs like PCB-77 ^36^.

**Figure 1.**
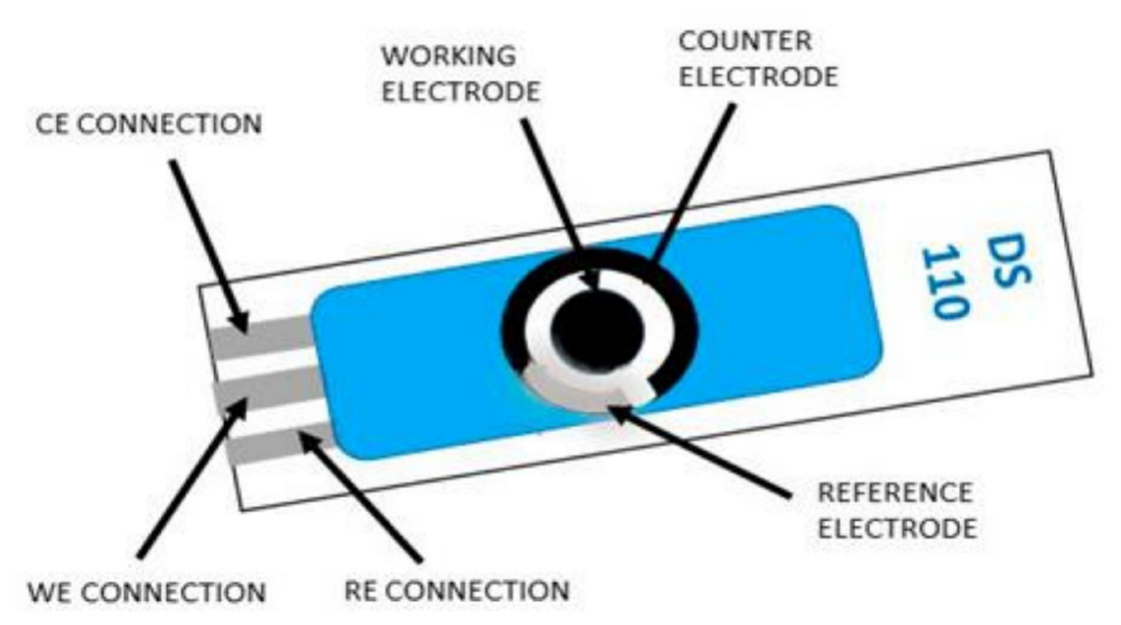
Common configuration of the screen printed electrodes (SPEs) (reproduced with permission from Michu *et al*. ^39^).

Towards miniaturization of the sensors to achieve on site detection and also reduce the amount of chemicals used as well as reduce the time required to process the traditional electrodes, screen printed electrodes (SPEs) have been adopted as substitute for the three electrode system in an electrochemical cell ^28^. The electrodes are produced by screen-printing technology and consists of circular working electrode (3 to 4 mm diameter) made of gold or carbon, a reference and a counter electrode made of silver as shown in Figure 1 ^38, 39^. The size of the electrodes means less sample volumes and polymerization solution in small volumes of up to 50 µL are applied as droplets thus minimizing on consumption of chemicals of which sometimes may be expensive or even toxic. Just like the normal electrodes, the working electrode can be modified for a wide range of applications. The only reported SPE based electrochemical sensor for PCBs used tin disulfide and cyclodextrin to modify the working electrode achieving detection of PCBs at micromolar range while demonstrating the stability of the electrodes for one week ^40^. The cost for production of SPEs is low offering the advantage of disposal after use, thus eliminating the electrodes regeneration steps that are associated with the use of chemicals ^41^.

## Bioelectrochemical sensors

Bioelectrochemical sensors utilize biologically generated recognition elements that include enzymes, antibodies and aptamers (short, single-stranded DNA or RNA molecules) that can selectively bind to a target with high affinity and specificity as the sensing layer ^42^. Biosensors portray high selectivity and specificity of the target analytes because of the specific nature of production ^18^, thus being able to detect analytes in complex matrices and minimizing the sample preparation steps. However their application is narrow due to their unstable nature in extreme experimental conditions, inability to be reused and the cost of production of the recognition element ^43^. A highly selective biosensor for detection of Aroclors that utilized anti-PCB antibody as the recognition element reported a method detection limit ranging from 0.39 to 3.3 ng mL ^-1^. Its applicability in environmental monitoring was supported by negligible cross reactivity of less than 3% for closely related chlorinated phenolic compounds and recoveries ranging from 100 to 106% for spiked environmental samples and 92 to 102% for the soil standard reference material (SRM) ^44^. A combination of silver nanoparticles with anti-PCB antibody as recognition element produced a sensor with even lower limit of detection of 6.3 × 10 ^-2^ ng mL ^-1^ for PCB 28 ^45^.

Miniaturized bioelectrochemical sensors using screen printed electrodes modified with enzymes, antibodies and antibody coated magnetic beads showed that PCBs can be detected in environmental samples at low concentrations giving results similar to the conventional HRGC-LRMS ^46– 49^. An electrochemical sensor based on carbon screen-printed disposable electrodes modified with antibodies was able to detect Aroclor 1242 at a detection limit of 4.6 µg mL ^-1^ ^48^. Further modification of the sensor by using magnetic beads coated on antibodies afforded a sensor with lower detection limits of 0.4 ng mL ^-1^ being able to quantify Aroclor 1248 in spiked marine sediment extracts and soil samples ^46^, PCB 28 as well as Aroclor 1242 and 1248 in milk samples at spiked concentrations of 5 ng mL ^-1^ and 0.1 µg mL ^-1^, respectively ^49^. Another sensor for environmental application was reported to be responsive to Aroclors 1242, 1248 and PCB 28, 52, 101, 118, 138, 153 and 180 in different organic matrixes at recoveries between 95% and 104%, with the results comparable to those of the standard HRGC-LRMS method ^47^.

Modifications using aptamers as recognition elements has seen sensors that are applicable for ultrasensitive detection of PCBs being reported. A highly selective aptasensor based on PCB-77 aptamer covalently bonded to nickel hexacyanoferrate nanoparticles/reduced graphene oxide (NiHCF-NPs)/rGO) hybrid as the recognition element demonstrated high selectivity and sensitivity reporting a limit of detection of 0.22 ng L ^-1^. Application of the sensor in determination of PCB -77 in spiked water sample gave recoveries of 88.0%, which were in the same range as those of HPLC at 104.1% ^50^. Most recently, Yuan *et al*. modified boron-doped diamond (BDD) coated with gold nanoparticles (Au-NPs) electrode with aptamers to come up with a sensor that detected PCB-77 at femtomolar concentration, demonstrating a limit of detection of 0.32 fM and recoveries up to 106% for water samples. The sensor was superior to all other sensors by being selective, reusable and being able to detect very low concentrations of PCBs in femtomolar range ^32^. Thus, a nanocomposite of BDD and gold nanoparticles coupled with the selectivity of aptamers provided a sensor that can be utilized for trace detection of PCBs. Au electrodes come with the advantages such as high electrical conductivity, chemical stability, and easy attachment to aptamers through Au-S bond formation ^51^, while BDD is characterized by chemical inertness, wide potential window, low background current, and good electrochemical stability ^52^.

## Biomimetic electrochemical sensors

**Figure 2.**
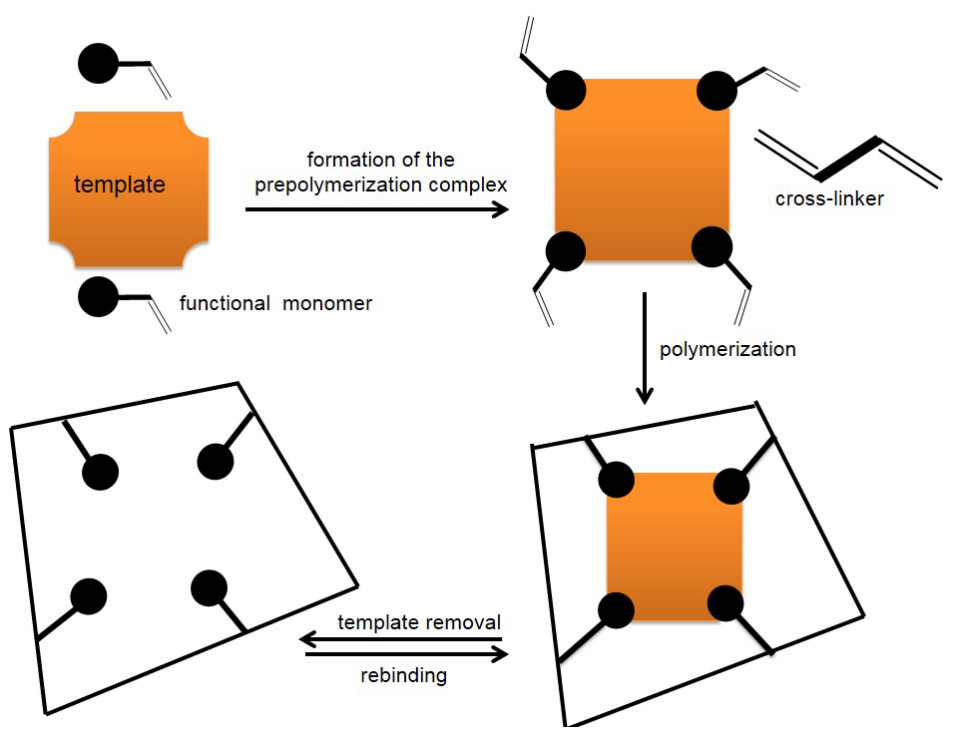
Schematic representation of the imprinting process (reproduced with permission from Ndunda, 2016 ^61^).

Biologically generated recognition elements in biosensors present a challenge in their production and also stability. They need to be generated from biological systems making them expensive to produce and are only stable at given temperature and pH ranges ^43^. In such circumstances, molecularly imprinted polymers (MIPs) that are able to mimic biological receptors in terms of specificity offer the best alternative in providing MIP based electrochemical sensors or biomimetic sensors. These polymers are able to achieve the high selectivity desired for recognition elements in sensors since they are tailored to display such selectivity ^53^. To attain these requirements, MIPs are synthesized by carrying out polymerization of functional monomers in the presence of the target analyte (called template in molecular imprinting) followed by template removal to leave behind a memory effect. It is envisaged that cavities that are complementary to the template are formed and therefore the template is able to rebind with high selectivity similar to that of natural receptors ^54, 55^. Figure 2 illustrates the various steps followed in molecular imprinting to generate MIPs.

The potential for integration of MIPs into sensors for environmental and biomedical applications has been discussed by Lowdon *et al.* and Kadhem *et al* ^37, 56^. In fabrication of such biomimetic sensors, the functional monomer used should be electropolymerizable and able to interact with the template through hydrogen bonding, electrostatic or π-π interactions ^34^. Methacrylic acid, pyrrole, o-aminophenol, 4-vinyl pyridine are examples of functional monomers that have been reported ^56^. Besides the template and the functional monomer, nanoparticles and carbon nanotubes that are characterized by high surface area, high electrical conductivities and electron transfer rates have been incorporated to increase the sensitivity of MIP-based sensors leading to detection limits in picomolar range ^43^.

MIPs have not been applied before as recognition elements to develop sensors for PCBs, but of interest is the most recent application to develop biomimetic sensor for chlorpyrifos; molecules that are chlorinated like PCBs. The sensors attained detection limit in femtomolar owing to the enhanced selectivity from MIPs ^57^, reporting results similar to those of the earlier reported aptasensor for PCBs ^32^. MIP-based screen printed electrode sensor for atrazine, another chlorinated molecule, has also been reported with detection limit of 0.4 µM ^58^. A highly selective MIP-based sensor for chloramphenicol that combined MIPs with carbon nanotubes reported a detection limit of 10 nM ^34^. The results of these particular articles demonstrate the potential for biomimetic sensors in ultra-trace level detection of the environmentally relevant pollutants. Prior to detection, pollutants that occur in very low concentration in the environment need to be extracted or preconcentrated and MIPs due to their selectivity have been shown to be suitable for that purpose ^34^. Therefore, a combination of MIP-based preconcentration step followed by detection using MIP-based sensors is a promising pathway that may be explored as a way of maximizing on these selective polymers to achieve the long term goals of ultra-trace level detection of PCBs.

The application of MIPs in sensors going forward is promising if their design can incorporate molecular modelling to select the appropriate functional monomers that provide high quality systems ^59^. Cleland *et al*. was able to model MIPs for PCBs showing that the results obtained are similar to those obtained in the laboratory ^60^. Molecular modeling combined with miniaturized screen printed electrodes will lead to fabrication of sensors that are affordable, selective and sensitive to target analytes, in addition to embracing the spirit of green chemistry. Such approach has been reported in design of Azithromycin sensor that was able to detect this pollutant in environmental samples at a detection limit of 0.08 µM ^34^.

## Conclusions

This review has shown that electrochemical sensors are potential alternatives to chromatographic instrumentation techniques as they are able to achieve detection of PCBs at trace-level concentrations by providing results that are comparable to the gold standard detection techniques. Sensors that do not achieve trace-level detection can be utilized in screening of PCBs before using the costly instrumentation. Modification of the electrochemical sensors using the biological elements or MIPs as alternatives as well as nanoparticles renders sensors that are highly selective and sensitive to the target analytes. Even more attractive are the disposable electrodes and portable potentiostat that promise affordable sensors through miniaturized systems for routing monitoring of PCBs. In this regard, a combination of rationally designed MIPs, nanomaterials, screen printed electrodes and portable potentiostat will lead to a sensor for detection of PCBs that meets the requirements of sensors including selectivity, sensitivity, remote usage and affordability.

## Data Availability

No data are associated with this article.
