# Supplementary material for: Towards miniaturized electrochemical sensors for monitoring of polychlorinated biphenyls
Source: Open Res Afr. 2023 Apr 14;6:5. [Version 1] doi: 10.12688/openresafrica.13983.1 (PMC10192943; doi:10.12688/openresafrica.13983.1)
Supplement: Peer review history

# Peer review: Reviewer response for version 1

**DOI:** 10.21956/openresafrica.15139.r29588

**Published:** 2023-05-17

**Authors:** Joshua M. Sila, Immaculate Michira

**Affiliations:** 1 Department of Chemistry, University of Nairobi, Nairobi, Kenya

- The authors have given a good review of the existing electrochemical sensors for PCBs.
- The title is appropriate and the literature cited is relevant for the review.
- The work is clearly and accurately presented with up to date references.
- The study design is appropriate and the review is well-detailed and informative.
- The conclusion drawn is supported by the results in the review.

Is the review written in accessible language?

Yes

Are all factual statements correct and adequately supported by citations?

Yes

Are the conclusions drawn appropriate in the context of the current research literature?

Yes

Is the topic of the review discussed comprehensively in the context of the current literature?

Yes

Reviewer Expertise:

Electrochemistry

We confirm that we have read this submission and believe that we have an appropriate level of expertise to confirm that it is of an acceptable scientific standard.

# Peer review: Reviewer response for version 1

**DOI:** 10.21956/openresafrica.15139.r29587

**Published:** 2023-05-03

**Authors:** Fredrick Okumu

**Affiliations:** 1 School of Biological, Physical, Mathematics and Actuarial Sciences, Jaramogi Oginga Odinga University of Science and Technology, Bondo, Siaya, Kenya

The review article is well-written and covers all aspects related to the miniaturized electrochemical sensors for monitoring polychlorinated biphenyls (PCBs). The draft has been prepared in line with the expectations of a review paper. The writing and content have both conformed to the expected scope and format of the publishing journal. 

 I am convinced that the article is scientifically sound and should be accepted for indexing as is with few areas of minor touches like the typos in the abstract and conclusion which should be **routine monitoring** and not ***routing monitoring***.

Is the review written in accessible language?

Yes

Are all factual statements correct and adequately supported by citations?

Yes

Are the conclusions drawn appropriate in the context of the current research literature?

Yes

Is the topic of the review discussed comprehensively in the context of the current literature?

Yes

Reviewer Expertise:

My areas of research are in electroanalytical chemistry and material chemistry.

I confirm that I have read this submission and believe that I have an appropriate level of expertise to confirm that it is of an acceptable scientific standard.
